# Somatic Drugs for Psychiatric Diseases: Aspirin or Simvastatin for Depression?

**DOI:** 10.2174/157015912800604533

**Published:** 2012-06

**Authors:** Juan Gibert Rahola

**Affiliations:** Department of Neurosciences, Faculty of Medicine, University of Cadiz, CIBER of Mental Health-CIBERSAM

**Keywords:** Amygdala, anti-inflammatory drugs, antidepressants, aspirin, BDNF, cytokines, depression, hippocampus, inflammation, MDD, NDAIDS, simvastatin, statins.

## Abstract

The evolution in the understanding of the neurobiology of most prevalent mental disorders such as major depressive disorder (MDD), bipolar disorder or schizophrenia has not gone hand in hand with the synthesis and clinical use of new drugs that would represent a therapeutic revolution such as that brought about by selective serotonin reuptake inhibitors (SSRIs) or atypical antipsychotics. Although scientists are still a long way from understanding its true aetiology, the neurobiological concept of depression has evolved from receptor regulation disorder, to a neurodegenerative disorder with a hippocampal volume decrease with the controversial reduction in neurotrophins such as BDNF, to current hypotheses that consider depression to be an inflammatory and neuroprogressive process. As regards antidepressants, although researchers are still far from knowing their true mechanism of action, they have gone from monoaminergic hypotheses, in which serotonin was the main protagonist, to emphasising the anti-inflammatory action of some of these drugs, or the participation of p11 protein in their mechanism of action.

In the same way, according to the inflammatory hypothesis of depression, it has been proposed that some NSAIDS such as aspirin or drugs like simvastatin that have an anti-inflammatory action could be useful in some depressive patients. Despite the fact that there may be some data to support their clinical use, common sense and the evidence advise us to use already tested protocols and wait for the future to undertake new therapeutic strategies.

## 1 INTRODUCTION

What would you think if you were a patient suffering from a depressive disorder and your doctor prescribed you aspirin or simvastatin and, when you asked him why, your doctor told you that the cause of your symptoms was an inflammation of the brain? You would possibly think that it is difficult to understand current advances in psychiatry. But this is neither a joke nor fiction as advances in the understanding of the neurobiology of true depression or major depressive disorder (MDD) have led us to consider that inflammation may play an important role in this disease. The evolution in the understanding of the neurobiology of the most prevalent mental disorders such as MDD, bipolar disorder or schizophrenia has not gone hand in hand with the synthesis and clinical use of new drugs which would represent a therapeutic revolution such as that brought about by selective serotonin reuptake inhibitors (SSRIs) or atypical antipsychotics. Researchers should consider the current hypotheses on the neurobiology of depression to reach the inflammatory hypothesis of depression and the possible usefulness of anti-inflammatory drugs such as aspirin and other drugs like simvastatin.

## 2 STATE OF THE ART

### 2.1 The Neurobiology of Depression

Although, today, it is accepted that MDD is a chronic, progressive and recurrent process related to structural and functional changes in the brain [1-4], understanding of its physiopathology, despite its prevalence, is infinitely below that of other chronic pathologies such as type 2 diabetes, hypertension or atherosclerosis. This is due to the fact that observation of pathological changes in brain is much more difficult than in the case other organs: techniques for studying the brain are based on post-mortem (with their many limitations) or neuroimaging studies that use indirect markers of activation and to the fact that the experimental models also give us information, the problem being the extrapolation of the results to human beings [5]. Furthermore, the majority of depressions (depressive disorders) is idiopathic and the limited knowledge of their aetiology is reflected in the risk factors: stressing events, endocrine alterations, cancer and undesirable side effects of drugs, among others, [6-8]. Genetic linkage studies are not conclusive and no responsible genes, permitting us to reproduce the disease in animals [8, 9, 10], that have been identified; genetic predisposition interacts with environmental risk factors and can trigger off depressive episodes in some patents [11].

Diagnosis of MDD is subjective and is based on the evaluation of specific symptoms affecting functioning over a certain period of time and these criteria overlap with other processes such as anxiety disorders [12, 13].

### 2.2 Neural Circuits and Depression

Different regions and circuits of the brain control emotions, reward and executive function. Changes in the function of limbic regions that have many interconnections [14], have been implicated in both depression and the action of antidepressants (ATDs). In post-mortem [7, 15] and neuroimaging [7, 16] studies decreases in grey matter and glial density have been observed in prefrontal cortex (PFC) and hippocampus, areas responsible for the cognitive aspects of depression, although the results are not consistent. Neuroimaging studies carried out with fMRI (functional magnetic resonance imaging) or PET (positron emission tomography) show that the amygdala and the subgenual cingulate cortex (Cg25) are correlated with dysphoric emotions [7, 13] while deep brain stimulation of white matter tracts around Cg25 and in nucleus accumbens is effective in resistant patients [17, 18]. These prosencephalic tracts, apart from controlling alertness and consciousness, modulate the importance of emotional stimulus and, in turn, are modulated by monoaminergic projections from mesencephalic and brainstem nuclei [6].

**Fig. (1):**
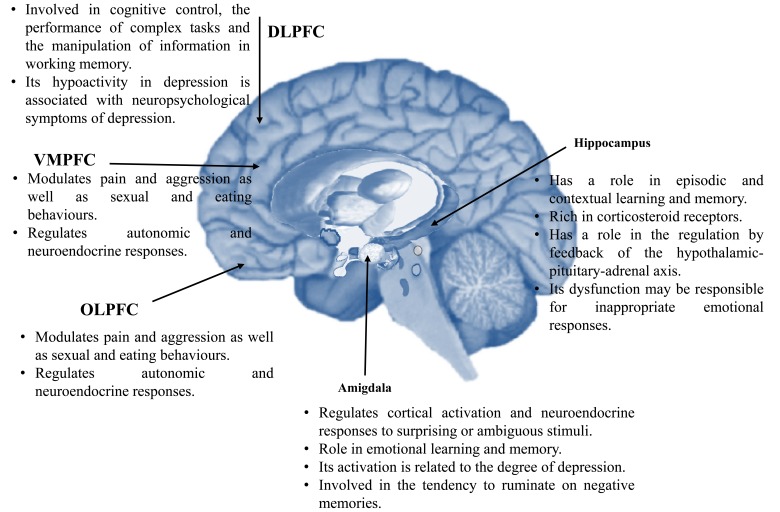
Brain areas involved in the regulation of mood.

The main areas of the brain involved in the regulation of mood are (Fig. **1**. Adapted from ref. no. [19]):

- The ventromedial prefrontal cortex (VMPFC) [20, 21]:
  - Modulates pain and aggression as well as sexual and eating behaviors.
  - Regulates autonomic and neuroendocrine responses.

- The lateral orbital frontal cortex (OLPFC) [22, 23]:
  - Activity increases in depression, obsessive-compulsive disorder (OCD), post-traumatic stress disorder (PTSD) and panic disorder.
  - Corrects and inhibits maladaptive, perseverative and emotional responses.

- The dorsolateral prefrontal cortex (DLPFC) [24]:
  - Involved in cognitive control, the performance of complex tasks and the manipulation of information in working memory.
  - Its hypoactivity in depression is associated with neuropsychological symptoms of depression.

- The amygdala:
  - Regulates cortical activation and neuroendocrine responses to surprising or ambiguous stimuli [25].
  - Role in emotional learning and memory.
  - Its activation is related to the degree of depression [7, 23].
  - Involved in the tendency to ruminate on negative memories.

- The hippocampus:
  - Has a role in episodic and contextual learning and memory [26].
  - Rich in corticosteroid receptors [27, 28].
  - Has a role in the regulation by feedback of the hypothalamic-pituitary-adrenal axis [25].
  - Its dysfunction may be responsible for inappropriate emotional responses [29].

Having reviewed the main areas involved in the neurobiology of depression we shall carry out a brief analysis of the role of monoamines in this disorder.

### 2.3 Monoamines and Depression

Several lines of evidence suggest an involvement of the neurotransmitter serotonin (5-HT) in the pathophysiology or pathogenesis of MDD [30] and that the noradrenergic or dopaminergic dysfunction would have a secondary role. However, it is more plausible that the functional imbalance between serotonin and noradrenalin (5HT/NA) be responsible for the inadequate functioning of certain brain nuclei and areas that would be reflected in certain symptomatic groups:

- Alterations of cortex regulation: concentration and memory alterations and an uncontrollable feeling of worry or guilt.
- Hypothalamic dysfunction: changes in appetite, libido, and autonomic symptoms.
- Thalamus and brain stem: sleep and activation alterations.
- Alteration of the tracts connecting the cortex to the hippocampus and the amygdala: chronic hypersensitivity to stress and fear that determines the characteristics of anxiety, anhedonia, aggression and lack of affective control.

However, it is possible that this monoamine deficiency has only a marginal contribution to genetic vulnerability [31, 32] since the depletion of amines has no effect on normal subjects [33] and, in experimental studies with animals, the dopamine (DA) and noradrenalin (NA) increase only produces a maladaptive effect in paradigms related to stress, as it strengthens the memory of adverse vital events [33, 34].

Moreover, acute monoamine increases due to ATDs induce medium-term secondary neuroplastic changes, which involve transcriptional and translational changes, mediating molecular and cellular plasticity [6, 36]. For example, 5-HT1B receptors interact with the p11 protein, a calcium binding protein, which increases in the cerebral cortex after chronic treatment with selective serotonin reuptake inhibitors (SSRIs) and decreases in the cingulate cortex of depressive patients [37]. Moreover the specific transgenic over-expression of p11 in the brain produces an antidepressant phenotype, which means that p11 increase mediated by SSRI is an important mechanism after the activation of the receptor [36].

Chronic administration of ATDs also up-regulates cAMP response element-binding (CREB) transcription factor in the hippocampus (taking part in the signal cascade resulting from the stimulation of different 5HT receptors and other receptors linked to G stimulating proteins) [35]. Furthermore, the activation of CREB in nucleus accumbens due to stress triggers off depressive responses, which emphasizes the crucial specific regional actions of neurotransmitters and their postsynaptic effectors which are not contemplated in the simplistic models [38].

Monoaminergic ATDs are still the basic treatment for depression, but their long-latency effect [39] and low remission rates have encouraged the search for more effective drugs [14, 40, 41].

### 2.4 Hippocampal Neuroplasticity: Neurotrophic Factors, Stress and Neurogenesis in the Adult Brain

Evidence suggests that depressive disorders are neurodegenerative disorders and there are studies providing information on the decrease in the number of neurons and glial cells in depressive patients. There is a decrease in the density of glial cells and the size of neurons in the DLPFC and caudal orbitofrontal cortex of depressed patients [42]. Other quantitative studies demonstrated a decrease in the density and size of neuronal glial cells in the anterior cingulate cortex and a low number of glial cells in the amygdala in MDD [43-45].

**Fig. (2):**
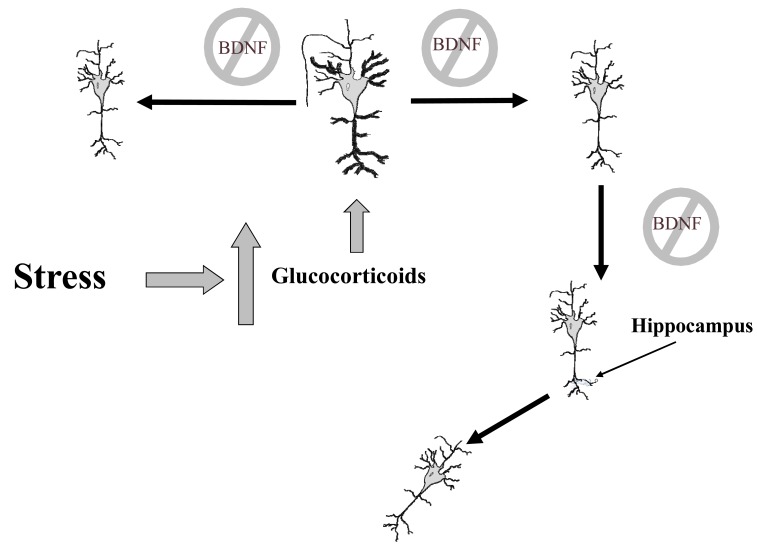
Stress, BDNF and apoptosis in hippocampus.

Among the most important MDD structural alterations is decreased hippocampal volume, which would be directly related to the duration of untreated depression and the antidepressants would partially block or would reverse hippocampal volume decrease [46-49]. Furthermore, the hippocampus is a key structure in the regulation of the hypothalamic-pituitary-adrenal axis observed in MDD [50], so that compromise of the hippocampus produces an alteration of neuroendocrine regulation which gives rise to high cortisol levels which can act as toxins in the body and these high levels of cortisol can also affect neuronal plasticity and survival through the modulation of a neuro- trophic factor, brain-derived neurotrophic factor (BDNF) [51] (Fig. **2**. Adapted from ref. no. [19]).

Decrease in the volume of the hippocampus and other prosencephalic structures in subgroups of depressed patients supports the hypothesis of decrease of neurotrophic factors in depression, especially the BDNF, which is profusely expressed in the adult limbic system. Studies with rodents indicate that stress reduces hippocampus-mediated signals, while chronic administration of ATDs increases them [6, 52]. Similar results have been found in the depressed hippocampus in human post-mortem studies [53] as well as an increase in plasma BDNF concentrations, whose origin is controversial [52]. The antidepressant effect of direct infusion of BDNF into rodent hippocampus [54] and its blockade in knockout animals without the gene that encodes this factor in prosencephalic regions, provide more direct proof of its causal participation in depression [55, 56].

The discovery of a possible relationship between the BDNF and MDD meant a great advance in the understanding of the neurobiology of depression. BDNF is a neurotrophic substance (it promotes the growth and survival of neurons) and there is considerable evidence that the levels of BDNF in the brain decrease during depression and, in theory, this could lead to atrophic brain changes, especially in the hippocampus [57].

The administration of ATDs normalizes the levels of BDNF, as has been observed both in studies with animals and in post-mortem studies on human brains of people suffering from mood disorders, whether they were taking medication at the time of death or not. Studies in humans comparing levels of serum BDNF in depressed patients to non-depressed subjects reveal significantly lower BDNF levels in depressed patients. It is most important to emphasize that depressed patients treated with antidepressants have similar BDNF levels to those of non-depressed subjects [58-60]. It has been demonstrated in animal models that some antidepressants (i.e. SSRIs, serotonin-norepinephrine reuptake inhibitors (SNRIs), tricyclic antidepressants (TCAs)), lithium and valproate increase BDNF levels in rat hippocampus. Typical antipsychotics can decrease BDNF levels while atypical antipsychotics can increase its levels [52, 61].

We could say that the hippocampus is the key to depression as both 5-HT and NE influence the balance of activity between excitatory (glutamatergic) and inhibiting (GABAergic) in PFC and limbic system. Excitatory neurons from the PFC have regulatory influence on the locus coeruleus and the dorsal nuclei raphe [51]. A combination of excessive excitatory input from the VMPFC and an increase in glucocorticoid levels caused by stress can have a toxic effect on the hippocampus. Modification of hippocampal function can contribute to a cognitive, emotional dysfunction and compromise neuroendocrine regulation in MDD [50].

Stress plays a very important part in the neurobiology of most depressive patients, although depression without stress can exist. There are data indicating that stress can trigger or aggravate depression and that chronic stress leads to the atrophy or remodeling of CA3 hippocampal neurons. Moreover, stress reduces hippocampal BDNF, which also reduces neurogenesis [52, 62].

A cellular effect of many (but not all) ATDs, including monoamine oxidase inhibitors (MAOIs), SSRIs, TCAs, SNRIs, noradrenergic and specific serotonergic ATDs, is the induction of adult hippocampal neurogenesis, the process by means of which the neural progenitors of the hippocampal subgranular zone (SGZ) divide mitotically to form new neurons, which differentiate and integrate into the dentate gyrus [36, 63]. In rodent and primate hippocampus, the adults generate neuronal cells, which arise from SGZ progenitor cells and migrate towards the granular cell layer where they differentiate into granular neurons [64].

Blockade of hippocampal neurogenesis slightly inhibits the "therapeutic" effect of the majority of antidepressant treatments in rodents [64] but antidepressant treatments increase the concentrations of different hippocampal growth factors that influence neurogenesis, possibly through the actions of CREB or other transcriptional regulators [6, 36, 64]. These include BDNF (which promotes neuronal survival) as well as the vascular endothelial growth factor (VEGF) and the VGF nerve growth factor inducible (its name is non-acronymic), which result in antidepressant and pro-neurogenic activities [65-67]. Activity dependent increases in neurogenesis could increase activity propagation in different hippocampal layers [68] and allow hippocampal networks to adapt and learn new experiences [69]. This would increase the possibility of the presence of intact neurogenesis during stressful episodes producing maladaptive learning and the appearance of depressive sequelae. While several types of stress reduce cell proliferation in the SGZ, decreased neurogenesis does not itself produce depression [69, 63]: Inhibition of hippocampal neurogenesis in rodents (through irradiation [70, 64] or genetic techniques [71]) does not produce anxiety or depressive behaviors. Taken together, these studies emphasize the weakness of a unified theory of depression: mechanisms which promote depressive symptoms in response to stress differ markedly between different neuronal circuits and can also be distinct from changes that underlie depression in the absence of external stress (endogenous depression). Moreover, neuroplastic processes that are required for antidepressant efficacy do not need to reverse the stress-induced alterations in plasticity and might function through separate and parallel circuits [5].

Neurogenesis is more prominent in sub-ventricular zones and in the hippocampal dentate gyrus SGZ. Early stressors may induce abnormalities in the development of amygdala, hippocampus, anterior cingulate cortex, corpus callosum and other structures, which play a critical role in responses to stress [72]. In adults, stress decreases neurogenesis in the hippocampal dentate gyrus subgranular zone [73] and environmental enrichment [74-76] and ATDs [77] have a positive effect on neurogenesis. The possible participation of BDNF has already been mentioned, although other factors may take part, since decrease has been observed in others such as FGF (fibroblast growth factor) [78] or NCAM (neural cell adhesion molecule), contributing to neurocognitive disorders in depressive processes related to stress [79].

The BDNF hypothesis is, however, too simplistic and not exempt from criticism. Many preclinical studies have been unable to demonstrate these changes produced by stress or ATDs or have obtained opposing results [80, 81]: conditional BDNF knockout show no depressive behavior [55], BDNF exerts a powerful pro-depressant action in the ventral tegmental area (VTA) and nucleus accumbens (its expression in nucleus accumbens is increased by stress [82], direct infusion into these zones increases behaviors related to depression [35, 83] and a selective knockout without the gene encoding BDNF in this circuit has an antidepressant effect) [82].

These data suggest that BDNF and its TrkB receptor produce different effects on behavior depending on the area of the brain [52].

### 2.5 Cellular Resilience and Depression

Although it might seem that this aspect should have been included in the previous section, we would like to give it special attention. Human responses to stress and adversity are very heterogeneous which means that some types of depression could be due to stressing events, or that the events moderately increase the risk of depression [1, 11]. However, reactive dysphoric states, as PTSD (post-traumatic stress disorder), only appear in about 10-20% of patients exposed to trauma [84]. Although there is a lot of data suggesting post-stress maladaptive neurobiological changes (decreased hippocampal neurogenesis and less BDNF) there is little information as to why most people adapt well (are resilient) to adversity [85]. In studies carried out on animals, analysing natural variations in the development of active escape in the learned-helplessness test, stress-induced upregulation of the transcription factor ΔFOSB (a stable, truncated protein product of the Fosb gene) in the midbrain periaqueductal grey nucleus was shown to promote a resilient phenotype. This effect was mediated through reducing expression of substance P, a neuropeptide released during stress [86].

Mesolimbic DA-mediated signalling would participate in emotional homeostatic mechanisms [37] so that vulnerability to social avoidance and other negative consequences of stress would be mediated by the increased excitability of VTA DA neurons and their subsequent increased activity-dependent release of BDNF onto nucleus accumbens neurons [86-88]. Resilient mice (with increased DeltaFOSB concentrations) [86] do not show this increase in VTA neuronal excitability due to the upregulating of voltage-gated potassium channels (molecular compensation to restore normal excitability and main maintain low levels of BDNF-mediated signalling in the nucleus accumbens).

Other possible mechanisms of resilience could be the release of neuropeptide Y from locus coeruleus nerve terminals onto the amygdala [85, 89]. There are studies which show stable individual differences in stress responses among genetically inbred mice, strongly implicating extra-genomic factors [36, 86, 90-92]. As these mice are kept in identical environmental conditions, the findings imply the importance of epigenetic mechanisms during development.

The study of gene expression of stress-vulnerable and stress-resilient mice revealed distinct transcriptional profiles in the VTA, nucleus accumbens [38] and hippocampus suggesting that the resilient behavior represents a different, active neurobiological process (not simply the absence of vulnerability [36]). The understanding of such molecular mechanisms of allostasis (efforts to maintain homeostasis) may be the way to the development of new drugs [93, 94].

### 2.6 Hippocampal-Neuroendocrine Interactions

In previous sections we have referred to the relationship of stress with depression [1, 11, 37, 50, 52, 62, 82, 86, 88, 89, 92-94], so that it is logical for alterations of stress axis hormones such as increased serum glucocorticoid concentrations to be produced in depression [95, 96]. On the one hand, we know that physical or psychological stress increases serum glucocorticoid concentrations and that their chronic administration can produce symptoms similar to depression [97] in rodents and, on the other, that excess glucocorticoids can reduce cellular proliferation in the hippocampal subgranular zone (SGZ) and produce atrophy in hippocampal sub-regions [93, 94], which may contribute to the reduction of its volume observed in depression. There are various facts supporting this relationship, such as that patients with Cushing’s syndrome have depressive symptoms and hippocampal atrophy [6, 93] and that some of the alterations of depression (insulin resistance and abdominal obesity) can be explained, at least in part, by the increase in glucocorticoids [98].

Hypercortisolaemia in depression is manifested at several levels:

- Alteration of glucocorticoid-receptor-mediated negative feedback [98].
- Adrenal hyper-responsiveness to ACTH [95].
- Hyper-secretion of Corticotropin*-*releasing hormone* (*CRH*), *originally named corticotropin-releasing factor (CRF) [99, 6, 100].

However, hypercortisolaemia only occurs in very severe depression [102] or accompanied by psychotic symptoms [6, 11] in which glucocorticoid antagonists show a certain amount of efficacy [102].

**Fig. (3):**
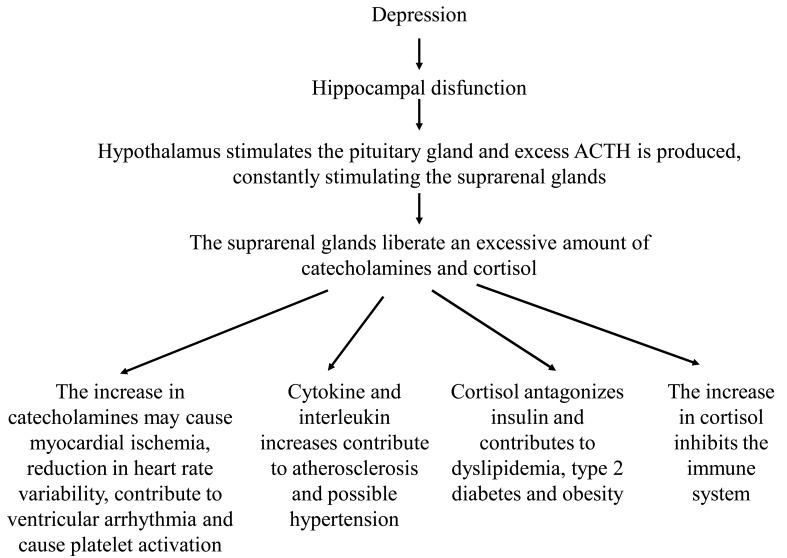
Hippocampal dysfunction and systemic consequences of the neuroendocrine alterations of depression.

In atypical depression (hyperphagia and hypersomnia) there would be hypocortisolaemia [101, 103, 104], which is also observed in fibromyalgia, chronic fatigue and PTSD [101]. High concentrations of glucocorticoids promote the mobilization of energy during stress while low glucocorticoid concentrations help the immune system against infection or physical injury [105]. It is important to point out that hippocampal dysfunction contributes to the neuroendocrine alterations of depression [6] and that these have many systemic consequences [106]: the hypothalamus stimulates the pituitary gland and excess ACTH is produced, constantly stimulating the suprarenal glands; the suprarenal glands liberate an excessive amount of catecholamines and cortisol and the increase in catecholamines may cause myocardial ischemia, reduction in heart rate variability, contribute to ventricular arrhythmia and cause platelet activation; cytokine and interleukin increases can also contribute to atherosclerosis and possible hypertension; finally, the cortisol antagonizes insulin and contributes to dyslipidemia, type 2 diabetes and obesity; the increase in cortisol also inhibits the immune system (Fig. **3**).

### 2.7 Inflammation, Neurogenesis and Neurodegeneration

Previous aspects had to be treated before we deal with this point. Several excellent reviews [107, 108, 109] have been recommended and, especially, a recently published editorial by Maes *et al. *[110]. However, out of all the works published there is one, Maes *et al. *[107], which establishes a very logical sequence to explain the inflammatory hypothesis of depression and which we have followed.

In previous sections we have tried to explain the data that strongly support the hypothesis that neurodegeneration would be added to a decrease in neurogenesis in MDD. Volumetric changes have been observed in hippocampus, amygdala, CPF, anterior cingulate and basal ganglia [111] as well as hippocampal cellular changes and neuronal and glial cell modifications [112]. Selective loss of hippocampal volume is due to both neuronal death and the decrease in neurogenesis [113].

How could we relate decrease in neurogenesis and neurodegeneration to inflammation? There are various, stimulating hypotheses and there is more and more information to support them [107-110]:

- Inflammation and neurodegeneration would play an essential role in depression.
- The increase in neurodegenerative processes could be due, at least in part, to inflammatory processes.
- Multiple pro-inflammatory cytokines, lesions induced by oxygen radicals, tryptophan catabolites and neuro-degenerative biomarkers are produced in depression.
- There are some vulnerability factors which predispose to depression, increasing inflammatory reactions:
  - Decrease in peptidase activity (dipeptidyl-peptidase IV, DPP IV)
  - Decrease in omega-3 polyunsaturated fatty acid levels.
  - Increased intestinal permeability
- The cytokine hypothesis considers that external (psychosocial) and internal (inflammations and postnatal period) could trigger off depression through inflammatory processes.
- Many ATDs have specific anti-inflammatory effects.
- While restoration of neurogenesis, which could be induced by inflammatory processes, would be related to the therapeutic efficacy of the ATD treatment.

Inflammation may produce oxygen radicals and this increase has also been observed in depression [115-117]. Oxidative stress is an important factor in neurodegenerative diseases [114]. It is responsible for programmed cell death, apoptosis, necrotic cell death and damage to DNA and membrane fatty acids which means that lipid signalling would be altered and lipid peroxidation increased. It would affect gene expression and proteolysis contributing to neurodegeneration [114-118]. Furthermore, antioxidant enzymes (dismutase superoxide, catalase and glutathione peroxidase) show therapeutic efficacy in neurodegenerative models [120, 121].

Another important factor is nitrosative stress. One of the modifications caused by nitric oxide metabolism imbalance is S-nitrosylation of protein cysteine residues [122]. Overproduction of NO may compromise neural energy and lead to neurodegeneration [122]. It inhibits cellular respiration and superoxide anions would be released, interacting with NO superoxide anions produced by the mitochondria producing peroxynitrite which is a powerful oxidant causing neurotoxicity [123].

Another common characteristic shared by depression and inflammation is IDO (indoleamine 2,3-dioxygenase) activation, an enzyme that induces the catabolism of tryptophan into TRYCATs (tryptophan catabolites along the IDO pathway), such as kynurenine with the consequent increase in TRYCATs [124-126]. Some TRYCATs, such as quinolinic acid and kynurenine, are extremely neurotoxic [127, 128], as quinolinic acid causes acute tumefaction and the destruction of post-synaptic elements, induces nerve cell degeneration, including hippocampal cell death and selective necrosis of granular cells, among others. Quinolinic acid causes a dose-dependent decrease in cholinergic circuits and may empty dopamine, choline, GABA and encephalin deposits [129-133]. The areas most affected by the neurotoxic effects of quinolinic acid would be the striatum, the pallidal formation and the hippocampus [134]. The neurotoxic effects of quinolinic acid may be due to:

1. Agonism at NMDA receptor level [135, 136]
2. Pro-oxidant effect through the formation of ferrous quinolinate chelates inducing lipid peroxidation
3. Exacerbation of the neurotoxic effects of corticosterone and IL-1 (interleukine-1) [135, 137].

According to Maes *et al. *[107], omega-3 polyunsaturated fatty acids (ω3 PUFAs) influence neurogenesis [138] through their anti-inflammatory and serotonergic effects and those they have on neurotrophins [139] in the following way:

1. ω3 PUFAs reduce the production of pro-inflammatory cytokines such as TNFα (tumor necrosis factor-α) and IL-1β.
2. ω3 PUFAs modulate membrane protein quaternary structure as well as their fluidity, which determines serotonin fixation [138]. Serotonin, on its part, stimulates neurogenesis [139].
3. ω3 PUFAs influence the levels of neurotrophins such as BDNF [141].

Cytokines are important modulators of mood, their SNC receptors being activated by both those produced centrally and peripherally [142]. Maes *et al.* [107] have recently proposed that the relationship between pro-inflammatory cytokines and depression would be based on 6 points:

1. In depression there is an increase in pro-inflammatory cytokines (interleukine-1β (IL- 1β); IL-6, and IFNγ (interferon*-*gamma)) and the acute phase of depression is due to high levels of pro-inflammatory cytokines such as IL-6 and IL-1β [144-146].
2. They are able to cause depressive-like behaviors [143].
3. They may explain the multicausal aetiology of depression by which psychosocial stressors and internal stressors (medical diseases) may trigger depression [146].
4. They may explain the serotonergic alterations of depression [148, 145].
5. The may explain alterations of the hypothalamic-pituitary-adrenal axis in depression [149]
6. Antidepressants block pro-inflammatory cytokines [150, 151]

However, studies directed towards relating depression to serum cytokine increases are inconsistent [152] and this might suggest that immunological activation would only be produced in depressive subgroups, especially when there is comorbidity with autoimmune process (rheumatoid arthritis) in which generalized inflammation may increase the risk of cardiac arrest as well as producing depressive symptoms [153].

**Fig. (4):**
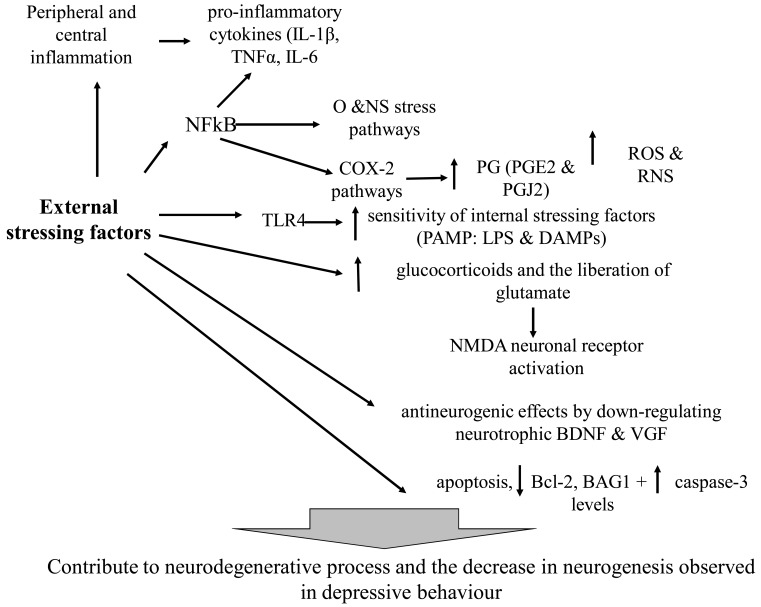
External and internal stress, inflammation and cell damage.

Could we, in a quite concise manner, relate external (or internal) stressors to inflammatory processes and cell damage, which occur in depression? External stressing factors in animals, such as mild chronic stress and learned-helplessness, are accompanied by behavior that we interpret as being depressive. These behaviors are accompanied by peripheral and central inflammation, with increased levels of pro-inflammatory cytokines, such as IL-1β, TNFα) and IL-6. External trigger factors and cytokines may induce NFkB (nuclear factor kB), which in turn induces the expression of pro-inflammatory cytokines; O &NS (oxidative, and nitrosative) stress pathways and COX-2 (cyclooxygenase) pathways. Through these pathways stress may cause a higher number of ROS and RNS (reactive oxygen and nitrogen species), including O2 and NO, which results in peroxynitrite generation. COX-2 may generate PG (prostaglandins), such as PGE2 and PGJ2. External stressing factors also increase the expression of TLR4 (Toll-like receptors), raising the sensitivity of internal stressing factors, including PAMP (pathogen-associated molecular patterns), such as LPS and DAMPs (lipopolysaccharide, and damage-associated molecular patterns). External stressing factors increase glucocorticoids and the liberation of glutamate and, consequently, provoke NMDA (N-methyl-D-aspartate) neuronal receptor activation. External stressing factors cause antineurogenic effects by down-regulating neurotrophic factors such as BDNF and VGF. Finally, external stressing factors cause apoptosis, with low levels of Bcl-2 (B-cell lymphoma 2) and BAG1 (Bcl-2 associated athanogene 1), and an increase in caspase-3 levels. Cytokines, as well as O and NS stress, NFkB, COX-2, PGs (PGE2), excitotoxic glutamatergic effects, apoptosis pathways and the decrease in neurotrophic substances contribute to neurodegenerative process and the decrease in neurogenesis observed in depressive behavior [154] (Fig. **4**).

### 2.8 Anti-inflammatory Effects of Antidepressants

The evolution of the understanding of the neurobiology of depression means that the concepts and names of hypotheses are constantly changing. Until a short time ago scientists were talking about the "inflammatory and neurodegenerative hypothesis of depression" [98] and they included a group of processes among which were the oxidative and nitrosative stress pathways, pro-inflammatory cytokines, decrease in antioxidants, including zinc, coenzyme Q and glutathione, the formation of TRYCATs, decrease in ω3 PUFAs and the increase in glucocorticoid levels. This hypothesis, now known as "inflammatory and neuroprogressive", since the term "neuroprogression" is more appropriate to describe the participation of apoptosis and anti-neurogenic and neurodegenerative progresses than the term "neurodegeneration" [155].

The effect of antidepressants, antagonizing the effect or the liberation of pro-inflammatory cytokines has been known for some time [150, 151] and would justify their anti-inflammatory effect. There seems to be no doubt that the administration of antidepressants produces anti-inflammatory effects (see the reviews of Maes *et al.* [156] and Kubera *et al.* [154]) as it reduces the production of TNFα, IL-12 and IFNγ and increases that of one of the most important anti-inflammatory cytokines, IL-10. The majority of antidepressants, including ISRS, tricyclic, IMAO, lithium and even atypical antidepressants, such as tianeptine, have anti-inflammatory effects [157]. For a more detailed study of inflammatory process in depression see the reviews by Gardner *et* *al*. [158], Maes *et al*. [159], Maes [160], Maes *et al*. [161], Son *et al*., [162], Szewczyk *et al*. [163] and Zunszain *et al*. [164].

There are studies relating the effect of antidepressants to their action on cytokines. We shall only mention one of these studies [165] during which 100 patients (36 men and 65 women) were treated with escitalopram 10-20 mg / day for 12 weeks. Responders and non-responders were identified according the Montgmery-Asberg scale (MADRS). Cytokine levels were measured at the commencement and during weeks 4 and 12 of treatment and compared to cytokine levels in healthy volunteers ((n = 45, 19 men and 26 women). The results obtained indicate that a higher level of TNF-α could predict the lack of response to treatment with escitalopram and that changes in sIL-2R concentration during the treatment were different in responders and non-responders.

Table **1** shows the main studies on the anti-inflammatory effect of ATDs.

## 3 ASPIRIN OR OTHER ANTI-INFLAMMATORIES FOR DEPRESSION?

This is the key point of our review and an aspect that may surprise the clinical psychiatrist. Should we administer NSAIDS like aspirin for depression? How and when, in monotherapy, associated with antidepressants? Theoretically, if depression is an inflammatory disease, the administration of anti-inflammatories should be beneficial.

### 3.1 Aspirin

Observation of the effects of aspirin on the mood is not something new and it was proposed before the inflammatory hypothesis of depression as, in 1996 [200], it was observed, for the first time, that the administration of low doses of aspirin to patients about to undergo coronary angiography was associated with less anxiety, depression or guilt.

#### 3.1.1 Preclinical Trials

We shall only mention two studies with animals during which aspirin has been used to increase the effect of fluoxetine. The first one [201] evaluated the effect of the co-administration of acetylsalicylic acid (ASA, 45 mg / kg or 22.5 mg / kg) and fluoxetine (FLX, 5 mg /kg) in the chronic escape deficit model of depression. In this model, FLX needed 3 weeks to reverse behavioral changes. In this study it was observed that combined treatment of fluoxetine and ASA reverted the condition of escape deficit in 7 days, a partial effect being observed after 4 days, and was maintained after 14 and 21 days of treatment. ASA alone was ineffective and the effect of fluoxetine was significant only at 21 days. The aim of the second study [202] was to investigate whether aspirin can be used as an augmentation agent in fluoxetine treatment resistant depressive rats induced by chronic unpredictable mild stress (CUMS). In this study, the effects of CUMS regimen and antidepressant treatment were assessed by behavioral testing, hippocampal expression of COX-2 and PGE2. 4-weeks of fluoxetine treatment antagonized the behavioral changes in approximately 70-80% of depressive rats. This means that, 20-30% of depressive rats were resistant to fluoxetine. In the hippocampus of fluoxetine treatment resistant depressive rats, a significant upregulation of COX-2 level and PGE 2 concentration was produced. However, in these rats, adjunctive aspirin treatment significantly improved the depressive behaviors and downregulated the COX-2 level and PGE2 concentration in the hippocampus.

There is a very important study that provides data leading in the opposite direction [203]. Scientists know that cytokines produced by glial cells regulate the serotonergic and noradrenergic brain systems and activate the hypothalamic-pituitary-adrenal axis. Antidepressants increase the levels of p11, a specific protein that regulates experimental rat models and interacts with serotonin receptors. In order to obtain information about possible interactions of antidepressants, cytokines, p11 and non-steroidal anti-inflammatories (NSAIDS), the researchers carried out experiments on mice and reanalysed the STAR*D data [204]. Selective serotonin, citalopram and fluoxetine reuptake inhibitors increased p11 levels in rat frontal cortex, but co-administration of ibuprofen (IBU) or acetylsalicylic acid (ASA) blocks this increase. IBU decreases plasma citalopram levels. The administration of desipramine produced a small p11 increase, which was not affected by IBU or ASA. Anti- depressants related to increased p11 depend on the signalling of two cytokines (IFNγ and TMF-α). In a rat depression model IBU, ASA and acetaminophen impeded behavioral response to SSRI, but not to other types of antidepressants. During the STAR * D study, in the patients who took citalopram for 12 weeks, the remission was significantly lower if they were taking NSAIDS than if they were not (45% vs. 55%). The findings were similar when other analgesics were used (37% vs. 54%). There is a difference between serotonergic and noradrenergic antidepressants because SSRI increase cytokines, which increase p11, resulting in an antidepressant response. NSAIDS (and acetaminophen) inhibit passage to cytokine activation. STAR*D has shown that the worst antidepressant response is associated with the use of analgesics.

#### 3.1.2 Clinical Trials

There is only one study that confirms the clinical efficacy of aspirin in depression and it is an open study. Mendlewicz *et al. *[205], based on their group's preclinical trials, [200], published a study that demonstrated that the administration of ASA, 160 mg / day shortened the onset of the action of fluoxetine. Another study [206] included seventy-seven patients with MDD, divided into two groups. The first group, consisting of 52 patients, received fluoxetine 20 mg, and the second one, in addition to fluoxetine 20 mg, received 150 mg of ASA. The activity of antioxidative enzymes, copper-zinc superoxide dismutase (CuZnSOD, SOD1), catalase (CAT), glutathione peroxidase (GPSH-x) and the concentration of malonyldialdehyde (MDA) was determined in erythrocytes, and the total antioxidant status (TAS) was determined in the plasma. All parameters were measured before and after three months of treatment. The results obtained indicate a significant decrease in the activity of SOD1, CAT and GSHP-x, as well as in MDA concentration after the combined therapy. A significant TAS increase was also observed. The study demonstrated that combined therapy with fluoxetine and ASA is characterized by the same efficacy and clinical safety as fluoxetine monotherapy, but with an additional improvement of oxidative stress parameters in patients treated for depression.

### 3.2 Cyclooxygenase-2 Inhibitors

COX-2 is expressed and has important functions in the central nervous system. It interacts with neurotransmitters such as acetylcholine, serotonin and glutamate as well as participating in the regulation of immune mechanisms in the central nervous system and of the inflammatory response by prostaglandins, especially PGE2. COX-2 inhibitors have been used as coadjuvants in the treatment of schizophrenia [207, 208] and it is logical that, since they are the most powerful anti-inflammatories, they have been tried in the treatment of depression.

#### 3.2.1 Preclinical Studies

In one study, the object of which was to determine the effect of celecoxib on the expression of COX-2, the concentration of PGE2 and the depressive behavior of rats subjected to stress paradigms, the results showed that 21 days of unpredictable chronic stress induced depressive behavior and an increase in the expression of COX-2 and the concentration of PGE2 in the rat brain. Chronic treatment with celecoxib reduced depressive-like behavior and caused a dose-dependent reversion of the levels of expression of COX-2 and of the concentration of PGE2 in stressed rats. Celecoxib also improved the emotional state and reduced the expression of COX-2 and the concentration of PGE2 in untreated rats. Moreover, one single dose of celecoxib reverted the expression of COX-2 and the concentration of PGE2 but did not alter depressive behavior in stressed rats.

#### 3.2.2 Clinical Studies

The majority of clinical studies carried out with celecoxib are randomized, double blind trials. The first, comparing it with fluoxetine and placebo, demonstrated that it was superior to fluoxetine alone [210] and another study, with a similar design [211], showed that it was superior to reboxetine alone. However, in a randomized controlled study in patients over 70 years of age, neither celecoxib nor naproxen had beneficial effects on depressive symptoms [212]. More interesting is the case of an elderly woman with depression and acute cognitive impairment, refractory to different anti-depressants, which only responded to acute treatment with celecoxib, maintaining remission over a period of 5 years [213].

## 4 STATINS

Despite the fact that preliminary studies with lovastatin [214] had shown no significant alterations to the cognitive functioning, the same team of researchers studied the possible effect of simvastatin on the cognitive functioning in hypercholesterolemic adults [215]. 300 hypercholesterolemic adults between the ages of 35 and 70 were included in a randomized, double blind, clinical trial and the participants were assigned daily treatment with placebo, 10 mg simvastatin or 40 mg simvastatin for a period of 6 months. The effects were minimal and do not support that statins cause major alterations to cognitive functioning.

A meta-analysis investigated the association between statins and mood disturbances [216], finding eight works giving information on the effect of statins on one or more of the following states of mood: depression, anxiety, anger, hostility, fatigue, confusion and vigour in adults over 18. Three papers reported some evidence of a positive association with depression, whilst another reported a decreased incidence of depression and the remainder reported no association. Of the six papers that studied anxiety, only one reported a statistically significant decrease in the incidence of anxiety. Two out of six papers reported increased aggression with statin administration, with one study further indicating a stronger effect with lipophilic statins. Only one paper reported decreased hostility. The authors concluded that there is conflicting evidence of a relationship between statins and mood and recommend further investigation, especially with older, female and lower socioeconomic status patients. Another study was directed at the research of the association between statin use and depressive symptoms in older persons [217] and the influence of gender and medical co-morbidity. During this prospective, observational study, with 1,803 participants aged 55 or over, demographic reference data, cholesterol levels and medical-co-morbidities were controlled, statin use not being associated with depressive symptom scores in the whole sample overall. Post hoc analysis suggested that statin use might be associated with fewer depressive symptoms in women (p = 0.02) and more depressive symptoms in men, especially those with more medical co-morbidities (p = 0.04) and multiple drug use (p = 0.02). According to the authors, this study provides no solid evidence to support an overall association of statin use and depressive symptoms.

## 5 DISCUSSION

### 5.1 NSAIDS

The first evidence relating NSAIDS to depression arose from the undesirable psychiatric effects caused by some of them, such as the appearance of depressive symptoms on withdrawal of indomethacin [218], the increase in the undesirable effects of imipramine due to ASA [219] or various depressive manifestations after the administration of NSAIDS [220, 221].

After all that has been said up to now, what the clinician asks himself is whether there is sufficient evidence to use aspirin in the treatment of depression? For this clinicians should consider whether or not they have more effective drugs to combine with antidepressants and whether the benefits of administering aspirin or other NSAIDS exceed their drawbacks. As regards the first aspect, there are many therapeutic possibilities that have shown themselves to very effective and with fewer risks than NSAIDS [222]. And, as regards the second aspect, NSAIDS produce a marked increase in intestinal permeability that may lead to peripheral inflammation due to bacterial translocation [223]. Given that intestinal permeability is a new inflammatory pathway in depression, prolonged use of NSAIDS for the treatment of MDD may endanger the intestinal wall and increase bacterial translocation [224]. For this reason, the use of aspirin to boost the effect of antidepressants is, in our opinion, controversial.

On the other hand, clinicians should bear mind that not all the studies agree with the beneficial effects of NSAIDS and that, in some of them, antagonism of the effects of ATDs has been observed [225, 226]. All of this should make us proceed with extreme caution in the face of these supposed advances in the treatment of depression.

### 5.2 Statins

The case of statins is much more complex. First of all, the association of low cholesterol levels with depression, violence, anxiety and suicide [227] versus the anti-inflammatory action of statins [228]. Secondly, their possible use to prevent depressive episodes in determined cardiovascular patients [229] versus the possible induction of depressive symptoms, especially in women [216, 230].

Above all, if researchers were to ask any cardiologist what the actions of statins are, the majority will just answer that they are drugs used in patients with dyslipidaemias, some will know that they have an anti-inflammatory effect and very few are aware of their possible undesirable effects on mood. For this reason, it may seem paradoxical that simvastatin may be recommended for the treatment of depression because of their anti-inflammatory action or to prevent the risk of depression in cardiovascular patients [229].

Several studies have demonstrated an association between low cholesterol levels and depressive symptoms in that MDD and bipolar disorder patients have lower brain cholesterol levels in comparison to normal controls [227, 231]. Higher cholesterol levels have been observed in some studies after patients have received pharmacological treatment for major depression [232]. These results have led to speculation that low plasma cholesterol in patients with mood disorders is partially due to an effect dependent on the state of the depressive disease.

Although, on an experimental level, chronically administered simvastatin improves learning and memory in control but not in olfactory bulbectomized animals [Douma 200], the effects on humans are not clear.

If clinicians are looking for another justification for statin use in depression they could refer to the actions of BDNF [234]. In the nervous system, the proteolytic cleavage of pro-BDNF, a BDNF precursor, to BDNF through the tissue-type plasminogen activator (tPA)-plasmin pathway represents one mechanism that can regulate the action of BDNF. *In vitro* studies have demonstrated that statins can induce tPA and inhibit plasminogen activator inhibitor-1, the major inhibitor of tPA. It is therefore possible that statins could act through the tPA-plasminogen pathway to increase BDNF and achieve an antidepressant effect. The authors of this paper suggest that statins could be useful for patients with major depression with an abnormality in the tPA-plasminogen pathway or co-morbidities relating to cardiovascular disease. The problem is that the BDNF hypothesis has many weak points, as described in the corresponding section.

But, as indicted by While and Keen [216], the evidence relating statins with depression is controversial. A review of the existing literature gives us four studies which found no change in depressive symptoms with their use [214, 216, 236, 237], although some studies found small modifications in subgroups of patients [238, 239], while others found a decrease in positive affect and even a direct relationship with depression [240]. Finally, a large observational cohort study suggests that there is a relationship between continuous statin use and an improvement in depressive symptoms, but this is not the case with intermittent use [241].

Despite any data there might be in favor of the possible favorable effect of statins in depressive patients, that this is a similar case to that of aspirin. Even if dealing with resistant depressions, there are other, much more effective drugs that it would be more reasonable to associate with antidepressants than statins.

Table **2** includes the main preclinical and clinical studies with NSAIDS and statins in depression.

## CONCLUSIONS

We do not know what scientists will discover in the future as regards the neurobiology of depression or what new molecules will be available. As Shawn Hayley [242] says, there are many possibilities as we have many therapeutic targets. We now have some hypotheses and molecules in the "therapeutic arena" and must play with the risk / benefit relationship. We should be more prudent and think of the patient, not create any more problems for him than he already has and, in answer to the initial question, it is, in our opinion better not to tell him that depression is an inflammation of the brain (although it may be partly true) and, above all, not prescribe either aspirin or statins for antidepressant purposes as their risk / benefit relationship has not been proved. It is possible that, in the future, if the inflammatory hypothesis of depression is confirmed, new anti-inflammatory molecules, with fewer undesirable effects and possibly effective in these patients, may be synthesized.

## Figures and Tables

**Table 1.** Anti-inflammatory Effects of Antidepressants

Level		References
Preclinical study	In ex vivo cultures, TCAs highly significantly blocked the production of IL-1β, TNFα and IL-6 in human purified monocytes, the production of IFNγ and IL-2 in human lymphocytes	[150]
Preclinical study	Imipramine: CMS-exposed Wistar rats: ↓Con A-stimulated IL-1, IL-2 production; CMS-unexposed Wistar rats: ↔ Con A-stimulated IL-1, IL-2 production (splenocytes)	[166]
Preclinical study	TCAs, like clomipramine and imipramine, reduce the number of Th1 cells secreting IFNγ in rats with experimental autoimmune neuritis.	[167]
Preclinical study	Desipramine ↓ LPS-induced TNF-α levels ↑ LPS-induced IL-10 levels (plasma) Paroxetine, venlafaxine ↔ LPS-induced TNF-α, IL-10 levels (plasma)	[168]
Preclinical study	Desipramine: in bulbectomized Rats ↓LPS-induced TNF-α, IL-1β levels; Sham operated ↓ LPS-induced IL-1β levels; ↔ LPS-induced TNF-α (plasma)	[169]
Preclinical study	In animal models, antidepressants decrease inflammation-induced brain cytokine production and actions as well as depressive-like symptoms. Imipramine and fluoxetine: ↔ LPS-induced TNF-α and IL-β mRNA; (spleen)	[170]
Preclinical study	Tianeptine ↓ LPS-induced TNF-α levels ↔ LPS-induced IL-1β, IL-10 levels (plasma), ↓LPS-induced TNF-α, IL-β RNAm (spleen) . Tianeptine ↓ LPS-stimulated IL-1β, TNF-α, IL-6 mRNAs and a tendency for ↑ IL-10 mRNA in the hypothalamus without changes in the hippocampus and pituitary in rats	[171]
Preclinical study	Desipramine: CMS-exposed: ↑ Con A-induced IL-10 production; CMS-unexposed C57BL/6 mice: ↔ Con A-induced IL-2, IL-4, IFN-γ production (splenocytes)	[172]
Preclinical study	The effects of antidepressants on the production of IL-6 and TNFα are partly modulated by serotonergic mechanisms.	[173]
Preclinical study	Bupropion lowers production of tumor necrosis factor-alpha and interferon-gamma in mice	[174]
Preclinical study	Mirtazapine Single injection in NET-KO mice: Production stimulated by Con A (splenocytes): ↓ ↓ IL-6, ↓ IFN-γ, ↑↑ IL-4; in C57BL/6J mice: 7 days, ↓ ↓ IL-6, ↓ IFN-γ, ↑ IL-4, Single injection: ↓ IL-6, ↔ IFN-γ, ↑ IL-4	[175, 176]
Preclinical study	Amitriptyline ↓ LPS-stimulated IL-1β, TNF-α release in primary mixed glial cells and nortryptyline in microglia cells	[177]
Preclinical study	Imipramine, fluoxetine and reboxetine: ↓ IFN-γ-stimulated IL-6 release in microglia 6-3 cells	[178]
Preclinical study	Paroxetine: ↓ IFN-α-induced IL-β, TNF-α and ↑ IL-10 production stimulated by Con A+LPS (whole blood culture) Paroxetine Normalized IFN-α-stimulated IL-1β, IL-10 levels in the rat hypothalamus	[179]
Preclinical study	Desipramine: ↓ LPS-stimulated IL-1β, IL-6, TNF-α release in hippocampus-derived adult neuronal stem cells	[180]
Preclinical study	Clomipramine, imipramine: ↓ LPS-stimulated TNF-α levels, ↓ LPS-stimulated IL-1β, TNF-α mRNAs without any changes in the unstimulated gene expression in BV-2 microglia cells	[181]
Preclinical study	Desipramine: ↓ LPS-stimulated IL-1β, TNF-α mRNAs in the rat cortex Desipramine: ↔ LPS-stimulated IL-1β, TNF-α mRNAs in cortical mixed glia cells	[182]
Preclinical studies	In brain cell cultures, TCAs and SSRIs significantly suppressIL-1β, IL-6 and TNF-α production	[183]
Clinical study	In some but certainly not all studies, antidepressant treatments were shown to normalize the initially increased IL-6 plasma levels in depressed patients	[184-186]
Clinical study	A 6-week treatment with antidepressants significantly decreased the initial increases in the stimulated production of IFNγ, significantly decreased the initially elevated plasma concentrations of a number of APPs (acute phase proteins), such as C-reactive protein, haptoglobin and alpha 2-macroglobulin.	[187]
Clinical study	Antidepressants may suppress the increased plasma levels of various APPs in depression, e.g. haptoglobin, fibrinogen, C3C, C4 and alpha antitrypsin, showing that antidepressants have anti-inflammatory effects	[188]
Clinical study	Clomipramine, sertraline, and trazodone significantly reduced the IFN gamma/IL-10 ratio in nine healthy volunteers.	[151]
Clinical study	Antidepressant treatments have specific suppressant effects on cell mediated immune responses by decreasing the production of IFNγ and/or increasing that of IL-10, a major negative immunoregulatory cytokine	[151,189, 190]
Clinical study	Depressed patients had significantly higher pre-treatment levels of TNFα, which were significantly decreased during treatment with amitriptyline, a TCA, but only in responders to treatment.	[191]
Clinical study	SSRIs, like paroxetine, may prevent the development of depression induced by IFNα-based immunotherapy	[192]
Clinical study	In patients with MS and depression, treatment during 16 weeks with sertraline, an SSRI, significantly suppressed the stimulated production of IFNγ and depression scores as well.	[193]
Clinical study	Treatment during 6 weeks with antidepressants significantly attenuated the increased IL-12 levels.	[194]
Clinical study	Imipramine, a tricyclic antidepressant (TCA), and venlafaxine, a serotonin/noradrenaline reuptake inhibitor (SNRI), 5-hydroxytryptophan (5-HTP), the precursor of serotonin, and a combination of 5-HTP and fluoxetine, a selective serotonin reuptake inhibitor (SSRI) increased the production of IL-6 by stimulated peripheral blood mononuclear cells of depressed patients	[195]
Clinical study	After antidepressant treatment, the IFNγ/IL-4 ratio was significantly decreased in depressed patients, suggesting that antidepressants suppress the Th-1 arm of cell-mediated immunity significantly.	[196]
Clinical study	In another study, the same authors found that treatment with antidepressants reduced the IL-12 concentrations and increased the TGFα levels, explaining why the IL-12/TGFα ratio was decreased by treatment with antidepressants	[197]
Clinical study	Treatment with sertraline decreased IL-12, while increasing TGFβ.	[198]
Clinical study	Fluoxetine affords robust neuroprotection in the postischemic brain via its anti-inflammatory effect	[199]

**Table 2.** Studies with NSAIDS and Statins

Level		References
Clinical observation	Administration of low doses of aspirin to patients who were going to undergo coronary angiography was associated with less anxiety, depression or guilt	[200]
Preclinical study	Aspirin has been used to increase the effect of fluoxetine in the chronic escape deficit model of depression.	[201]
Preclinical study	Adjunctive aspirin treatment significantly improved the depressive behaviors and downregulated the COX-2 level and PGE2 concentration in the hippocampus in fluoxetine treatment resistant depressive rats induced by chronic unpredictable mild stress.	[202]
Preclinical study	Anti-inflammatory drugs in mice attenuate antidepressant effects of SSRIs.	[203]
Clinical study	During the STAR * D study, in the patients who took citalopram for 12 weeks the remission was significantly lower if they were taking NSAIDS than if they were not. The findings were similar when other analgesics were used.	[204]
Clinical: pilot open-label study	The administration of ASA shortened onset of action of fluoxetine.	[205]
Clinical trial	Combined therapy with fluoxetine and ASA is characterized by the same efficacy and clinical safety as fluoxetine monotherapy, but with an additional improvement of oxidative stress parameters in patients treated for depression	[206]
Preclinical study	21 days chronic unpredictable stress induced depressive-like behaviors and increased the COX-2 expression and PGE2 concentration in rat brain. Chronic treatments with celecoxib alleviated the depressive-like behavior and reversed the levels of COX-2 expression and PGE2 concentration in stressed rat in a dose-dependent manner.	[209]
Clinical trial	The combination of fluoxetine and celecoxib showed a significant superiority over fluoxetine alone in the treatment.	[210]
Clinical trial	The celecoxib group showed significantly greater improvement compared to the reboxetine-alone group.	[211]
Clinical trial	Celecoxib or naproxen treatment does not benefit depressive symptoms in persons age 70 and older	[212]
Preclinical study	Widely used anti-inflammatory drugs antagonize both biochemical and behavioral responses to SSRIs in mice.	[225]
Clinical study	NSAIDS and other analgesics inhibit SSRI efficacy in a clinical population.	[225]
Clinical study	The use of statins after a cardiac intervention is associated with reduced risk of subsequent depression.	[229]
Preclinical study	Simvastatin improves learning and memory in control but not in olfactory bulbectomized rats	[233]
Preclinical study	Statins may enhance the proteolytic cleavage of proBDNF: implications for the treatment of depression	[234]
Clinical studies	Statins: no effect on mood	[214, 235-237]
Clinical studies	Changes in older adults	[238, 239]
Clinical trial	Statins use related to depression	[240]
Clinical study	Statins long-term use related to psychological well-being	[241]

Rush AJ, Trivedi M, Fava M. Depression, IV: STAR*D treatment trial for depression. *Am J Psychiatry*. 2003 Feb; 160(2): 237.
